# Prevalence of Depression, Anxiety, and Stress among High School Students during the COVID-19 Pandemic: A Survey Study in Western Mexico

**DOI:** 10.3390/ijerph192316154

**Published:** 2022-12-02

**Authors:** Guillermo Alonso Cervantes-Cardona, Gabino Cervantes-Guevara, Enrique Cervantes-Pérez, Clotilde Fuentes-Orozco, Francisco José Barbosa-Camacho, Jonathan Matías Chejfec-Ciociano, Irma Valeria Brancaccio-Pérez, María Fernanda Zarate-Casas, Fanny Yesenia González-Ponce, Kriscia Vanessa Ascencio-Díaz, Mario Jesús Guzmán-Ruvalcaba, Tania Abigail Cueto-Valadez, Andrea Estefanía Cueto-Valadez, Alejandro González-Ojeda

**Affiliations:** 1 Departamento de Disciplinas Filosófico, Metodológicas e Instrumentales, Centro Universitario de Ciencias de La Salud, Universidad de Guadalajara, Guadalajara 44340, Mexico; 2 Departamento de Bienestar y Desarrollo Sustentable, Centro Universitario del Norte, Universidad de Guadalajara, Colotlán 46200, Mexico; 3 Departamento de Medicina Interna, Hospital Civil de Guadalajara Fray Antonio Alcalde, Calle Hospital 278, Guadalajara 44280, Mexico; 4 Unidad de Investigación Biomédica 02, Centro Médico Nacional de Occidente, Instituto Mexicano del Seguro Social, Guadalajara 44349, Mexico

**Keywords:** anxiety, depression, stress, high school, COVID-19

## Abstract

Social isolation and school closure may predispose adolescents to higher prevalence rates of depression, anxiety, and stress. In this cross-sectional observational study, the validated Spanish version of the Depression, Anxiety, and Stress Scale was administered to 3112 students aged 14–22 years old. We also collected data on participant gender, age group, school shift (morning or afternoon), school year, family type, whether they or any first-degree relative had been infected with COVID-19, whether any family member had died of COVID-19, and whether either of their parents worked. Mean scores were 8.34 ± 6.33 for depression, 7.75 ± 5.89 for anxiety, and 10.26 ± 5.84 for stress. Female students presented significantly higher scores on all three measures compared with male students. Students who had been infected with COVID-19, who had an infected family member, or who had a family member who died of COVID-19 also presented higher scores on all three measures. Identifying the symptoms and warning signs of depression and anxiety disorders is critical, particularly in vulnerable populations like adolescents.

## 1. Introduction

During the COVID-19 pandemic, social isolation and school closures may have exacerbated a variety of preexisting mental health concerns among adolescents and young adults. The lockdown may have resulted in eroded schedules, feelings of isolation or loneliness, fear of infection, and changes in sleep behavior, all of which can predispose a population to future mental health issues [1,2]. Some authors assert that these concerns will remain for months, if not years, after the pandemic has ended [3].

Many scales, including the Reynolds Adolescent Depression Scale [4], the Perceived Stress Questionnaire [5], and the Hamilton Anxiety Rating Scale [6], are now available for assessing adolescent stress, depression, and anxiety. The Depression, Anxiety, and Stress Scale (DASS-21) is a valuable self-report scale that can screen for anxiety, depression, and stress in one questionnaire.

Because this scale was originally designed for the adult population, numerous studies have been conducted to determine whether it can be used among those aged 14–22 years old [7]. Although there has been significant debate on this subject, some research has had positive outcomes. According to a study on adolescents from multiple nations (Chile, Australia, China, and Malaysia), DASS use in this group appears to be appropriate and valuable [8]. Several studies have also found that DASS is more effective for detecting stress, anxiety, and depression in adolescents and young adults [9,10,11,12].

One study reported that the abridged DASS (DASS-21) with a three-factor model has excellent reliability among adolescents. Another study of Vietnamese adolescents found that the DASS-21 is an effective tool for detecting symptoms of common mental health disorders, particularly depression and anxiety [13]. Other studies have shown that it is an effective instrument for detecting negative feelings in adolescents. However, the number of factors used among studies has been inconsistent, and some authors discourage use of this scale among young adults [7,9,10,11,12].

Incident mental health issues are increasing, and this rise is expected to continue [14,15]. During the COVID-19 pandemic, a worldwide survey of 1653 participants from 63 nations revealed significant differences in anxiety and stress levels between young people (18–35 years) and those over 36 years old [16]. Compared with pre-pandemic levels, a longitudinal study conducted in Germany with 6038 participants revealed that a greater proportion of subjects scored above the threshold for depression and anxiety on screening tests. Younger groups experienced greater distress than older groups. Symptoms of anxiety and depression increased most among people aged 18 to 29 [17].

A study carried out in Canada during the first wave of the COVID-19 pandemic found a slight increase in the prevalence of severe depression in a sample of 22-year-old young adults compared with their own assessment prior to the outbreak [18]. Another study conducted in the United States during the pandemic lockdown revealed an upsurge in depressive and anxious symptoms in adolescents and young people, particularly among women. This research concluded that the pandemic negatively impacted youth mental health [19].

In contrast, pre-pandemic, 7.8% of adolescents worldwide were expected to develop an anxiety disorder. The pandemic’s impact on students’ mental health has risen over time, with the prevalence of anxiety and depression gradually increasing to affect nearly one in every five children and young adults during the pandemic lockdown, as reported in mid-2021 [15,16]. A meta-analysis of the effect of COVID-19 on the mental health of the Mexican population revealed that 42% had an anxious emotional state, and 5.2% to 86.6% presented depressive symptoms. Among the examined publications, female gender and younger age came out as significant risk factors for mental health disturbance during the pandemic [20,21].

According to the findings of a pre-pandemic study [14] conducted on high school students in Mexico, 68% of females and 32% of males exhibited symptoms of depression.

During the first week of remote learning at a university in Mexico (13 March 2020), many of the students reported being fatigued (30%) and bored (9%). During the first weeks of the pandemic lockdown, 51% reported anxiety, and 86% of students reported depressive symptoms [22]. It is hypothesized that mental disorders will become more prevalent as a result of social changes resulting from the COVID-19 pandemic. The study objectives were to evaluate the prevalence of depression, anxiety, and stress among students in Guadalajara during the COVID-19 pandemic and to identify any differences in prevalence based on student gender, age group, school year, family composition, COVID-19 infection, COVID-19 infection of a first-degree relative, death of a family member from COVID-19, or whether either parent was employed.

## 2. Materials and Methods

### 2.1. Design

For this cross-sectional observational study, the recruitment team visited six high schools between May and July of 2021. A non-probabilistic method of purposive sampling was used to recruit participants. Students were invited to complete the survey after being informed of the purpose of the study and the nature of their participation. After receiving their consent, a link to the online survey was provided to the students. There was a 15 min time limit per classroom, and instructions were provided on how to complete the survey. We collected data on participant gender, age group, school shift (morning or afternoon), school year, family composition, whether they or any of their first-degree relatives had been infected by COVID-19, whether any family member had died of COVID-19, and whether either of their parents worked.

### 2.2. Sample

This study was conducted between May and July 2021. The required sample size was calculated using the Kelsey formula:

$$\frac{{\left(Z\alpha +Z\beta \right)}^{2}*p(1-p)(r+1)}{r{\left(p0-p1\right)}^{2}}$$

As noted above, before the pandemic, 7.8% of adolescents worldwide were expected to develop an anxiety disorder [23]. In contrast, recent studies have shown a 20.5% prevalence in anxiety disorders among adolescents during the pandemic [14], with an alpha error of 0.5 and a beta error of 0.90. Thus, we determined that a minimum sample size of 88 participants was required.

### 2.3. Instruments

The validated Spanish version of the DASS-21 was used [9,10]. This 21-item survey assessed three unique constructs: depression, anxiety, and stress. The core anxiety symptoms were trembling, increased heart rate, sweaty hands, and avoidance behavior. Depression symptoms were hopelessness, low positive affect, devaluation of life, and self-deprecation. The tension/stress subscale assessed tension, impatience, irritability, difficulty relaxing, and agitation. Each construct included seven scenario questions for which the participant needed to select the response option that best represented their feelings over the past week. Each four-point Likert scale response option ranged from 0 (“did not apply to me”) to 3 (“applied to me very much”). Cut-off scores for each construct are shown in Table 1. Internal validity was good for each subscale: depression (α = 0.92), anxiety (α = 0.89), and stress (α = 0.88).

### 2.4. Data Analysis

Statistical analyses were conducted using SPSS version 25 (SPSS Inc., Chicago, IL, USA). The descriptive analyses included proportions, means, and standard deviations. The Kruskal–Wallis test was used to establish significance. For quantitative variables, Student’s *t*-test was performed. A probability value of <0.05 was considered statistically significant.

### 2.5. Ethical Considerations

All procedures performed in studies involving human participants were in accordance with the ethical standards of the institutional and/or national research committee and with the 1964 Helsinki Declaration and its later amendments or comparable ethical standards. Informed consent was obtained from all individual participants involved in the study. The study protocol was registered under National Clinical Trials number NCT05190107.

## 3. Results

We included 3112 students aged 14–22 years (average age 16 years). Their demographic characteristics are in Table 2. As presented in Table 3, the participants’ mean score for depression was 8.34 ± 6.33, for anxiety 7.75 ± 5.89, and for stress 10.26 ± 5.84. Female students presented significantly higher scores for all three conditions (*p* < 0.001).

The sample was divided into two age groups: 14–16 years (n = 1635, 52.5%) and 17–22 years (n = 1477, 47.5%). The criterion was determined using a post hoc analysis of the sample to compare groups of a comparable size. Those aged 17–22 years had significantly higher stress scores than those aged 14–16 years (*p* = 0.015). There were no significant age group differences for the depression (*p* = 0.081) or anxiety (*p* = 0.571) scores.

An independent-samples t-test was conducted to compare students with morning or afternoon school shifts on the three conditions. Students who attended class in the morning had significantly higher depression scores than those who attended class in the afternoon (*p* = 0.035). There were no significant shift group differences for the anxiety (*p* = 0.143) or stress (*p* = 0.302) scores.

Students were also divided into two school year groups: Group 1 was first–third-semester students (n = 1658, 53.3%) and Group 2 was fourth–sixth-semester students (n = 1454, 46.7%). Group 2 students had significantly higher stress scores (*p* = 0.038). There was no significant group difference in depression (*p* = 0.373) or anxiety (*p* = 0.437) scores. When categorized by depression, anxiety, and stress severity, most students presented normal scores. However, 107 (3.4%) presented extremely severe anxiety. The comprehensive severity distributions are in Table 4.

A Kruskal–Wallis test examining the conditions between family composition types showed a significant difference (*p* < 0.001). The less nuclear-type the family, the more likely the student was to have higher scores for each of the three conditions. Students who had COVID-19 showed considerably greater scores for all three measures when compared with those who had not (*p* < 0.001). Comparing the students who had an infected family member and those with no infected family members, we found that the former scored higher on all three conditions (*p* < 0.001). Students who had a family member who passed away from COVID-19 only presented significantly higher mean scores for anxiety and depression (*p* = 0.006, 0.020), but not for stress (*p* = 0.103).

Finally, we analyzed scores between students whose parents were or were not at home because of work. Comparing students whose mothers had or did not have a job, the former scored significantly higher on stress (*p* = 0.006), depression (*p* = 0.004), and anxiety (*p* = 0.001). Comparing students whose fathers had or did not have a job, the later scored significantly higher on depression (*p* = 0.036), there were no group differences in the stress (*p* = 0.240) or anxiety (*p* = 0.051) scores.

## 4. Discussion

Mental health among students throughout the pandemic has become a marked global issue. The focus herein was on the psychological effects of the COVID-19 epidemic on high school students. Although the majority of our sample had normal depression, anxiety, and stress scores, over 23% had moderate-to-severe depression, and 35% had moderate-to-extremely severe anxiety.

Although our sample presented lower scores than those reported for global student samples [18,19], at least 15% of our participants showed signs of severe anxiety. This is important, as it represents a meaningful number of students and shows that mental health cases may have risen from persistent exposure to global or local news about the pandemic and added daily life stressors, in addition to hormone changes during puberty [16,20].

Our findings herein are consistent with those of others showing that female students have higher DASS-21 anxiety and depression scores compared with male students [12,21,22,24]. In a study from Australia, 18% of women reported at least moderate-level depressive symptoms, 33% reported at least moderate-level anxiety symptoms, and 16% reported at least moderate stress levels, whereas these values were 15%, 34%, and 12%, respectively, among men. In the same study, younger adolescent females had higher depression, anxiety, and stress scores than the male adolescents, although the difference was not statistically significant [25]. By contrast, while Rodrigo et al., did not find differences between 9th- and 10th-grade students, those in grade 11 had significantly greater scores of depression and severe anxiety [26]; these findings suggest a link between higher levels of education and stress, sadness, and anxiety scores, similar to our findings.

Herein, we discovered that students aged 17–22 years had elevated stress scores compared with those aged 14–16 years, although there was not a significant age difference in depression and anxiety ratings. When the DASS-21 and Impact of Event Scale-Revised (IES) were used to determine the incidence of mental health issues among high school students in China during the pandemic school closure, IES scores reflected trauma-level suffering among 22.7% of the sample. Furthermore, these participants’ DASS-21 score averages showed moderate depression and stress, and severe anxiety [27].

We found significant effects of family type on the three outcome measures, with students from less nuclear-type families more likely to have elevated stress, depression, and anxiety scores. According to Wang et al., family dysfunction had a significant, positive relationship with anxiety and depression. The quality of the home environment appeared to contribute positively to the development of adolescent self-esteem via the mediator of loneliness [28,29,30]. Furthermore, intrafamily relationship problems such as low empathy and conflict were found to predispose young Mexican adolescents to depression and difficulties with problem-solving compared with those in families with appropriate emotional expressions, unity, and empathy toward one another [31,32].

Furthermore, adolescents in families with lower levels of dysfunction may be able to express their thoughts and emotions more freely and effectively [33]. That study supported the notion that adolescent loneliness is exacerbated by familial turmoil, leading to higher levels of anxiety and despair. Because adolescents are more receptive to interpersonal contexts and less skilled at controlling their emotions, this link may be stronger during this developmental period than others.

Parental engagement is crucial. Greater peace and closeness at home contributes to better adolescent mental and physical health [26,27]. Constant exposure to information about mortality and disease appears to significantly impact mental health and propensity to exhibit depressive symptoms, at both regional and global levels [13]. Herein, we discovered that students in families with any COVID-19-infected member had higher scores on the three mental health factors compared with those in families with no infected member, and that those whose families lost a member to COVID-19 also had higher scores. Having a family member, relative, or friend infected with or die from COVID-19, impacted the fear response, creating higher levels of depression, anxiety, and stress compared with those without these experiences [34].

Our comparison of students whose father and/or mother worked and those whose did not showed that those with a working mother scored higher for all three factors, especially stress. Unexpectedly, those with a father who did not work had higher ratings, with a statistically significant difference for depression but not stress or anxiety. A topic for future research, this may ultimately be explained by paternal frustration, self-criticism, and rage, manifesting as violent, abusive, or neglectful child maltreatment. Because societal conventions create expectations that fathers will work outside the home, these outcomes occur more frequently in homes with an unemployed father than in those with an unemployed mother, resulting in child maladjustment [35,36,37].

We discovered that approximately one-fourth of the participants had moderate to severe depression, and nearly one-third had moderate to severe anxiety, making this research relevant. Prior to the COVID-19 pandemic, the prevalence of mood and anxiety problems in the region was much lower [38]. According to several studies, implementing educational programs and initiatives by their peers can improve program adherence and comprehension of mental health issues [39,40]. Because of the difficulties of conducting in-person therapy due to lockdowns and restrictions, digital interventions for mental health have been researched. Pedruzo et al. [41] reported that digital therapy, notably cognitive behavioral and dialectical behavioral therapy, effectively reduces anxious and depressive symptoms. Dialectical behavior therapy was found to be an effective digital intervention for reducing anxiety and depression symptoms in university students during the pandemic [42].

This study has some limitations. Initially, the validated Spanish version of the DASS-21 was administered, as there was no Mexico-specific version. In addition, despite our sample size, it was comprised of kids from a single high school in a specific region of Mexico, making it impossible to generalize our findings. We did not exclude pupils diagnosed with depression or anxiety previous to the pandemic, which may have been a complicating factor when evaluating our results. The research was undertaken prior to the creation of a COVID vaccination. As the COVID-19 vaccine has spread over the globe and preventative measures such as quarantine and social isolation are less prevalent than at the beginning of the pandemic, any replication of these procedures may change. Additional research is necessary to assess the long-term effects of this intervention on our youth.

## 5. Conclusions

A strength of the study is that DASS-21 provides straightforward scoring for assessing depression, anxiety, and stress symptoms. Once a precedent is created, this instrument can be used to examine the population over the long term and to compare the results of other studies employing the same questionnaire. These findings reinforce the notion that the impact of the pandemic on mental health is a recurring pattern across communities. Identifying the demographic variables associated with a decline in mental health should serve as a model for implementing health interventions focused on the at-risk population. Risk factors such as young age and female gender, as demonstrated in multiple studies, as well as a non-nuclear family, a history of COVID-19 infection, and the death of a relative due to COVID-19, should be emphasized among Mexican youth. Future research is essential to develop diagnostic tests and treatment interventions for this population’s illnesses, such as depression, anxiety, and stress. This will be guided by correctly identifying the numerous demographic characteristics linked with these conditions.

## Figures and Tables

**Table 1:** Depression, Anxiety, and Stress Severity cut-off scores.

Cut-off Scores	Depression	Anxiety	Stress
Normal	≤9	≤7	≤14
Mild	10–13	8–9	15–18
Moderate	14–20	10–14	19–25
Severe	21–27	15–19	26–33
Extremely Severe	>28	>20	>34

**Table 2:** Participants’ demographic characteristics.

Gender, n (%)	
Female	2101 (67.5%)
Male	1011 (32.5%)
Age (years)	
Mean ± SD	16.49 ± 1.04
Range	14–22
Semester	
1st Semester	593 (19.1%)
2nd Semester	662 (21.3%)
3rd Semester	403 (12.9%)
4th Semester	629 (20.2%)
5th Semester	203 (6.5%)
6th Semester	622 (20%)
Shift, n (%)	
Morning Shift	1744 (56%)
Afternoon Shift	1368 (44%)
Family Type, n (%)	
Nuclear family	1795 (57.7%)
Extended Family	616 (19.8%)
Single Parent Family	570 (18.3%)
Others	131 (4.2%)
COVID-19 Infection, n (%)	
Yes	252 (8.1%)
No	2860 (91.9%)
Infected Family Members, n (%)	
Yes	1769 (56.8%)
No	1343 (43.2%)
Family COVID-19 Survivors	
Yes	1520 (48.8%)
No	219 (7%)

**Table 3:** DASS-21 mean scores categorized by age groups, gender, scholar shift, and family characteristics.

Age Groups	Depression Mean Scores	*p*-Value	Anxiety Mean Scores	*p*-Value	Stress Mean Scores	*p*-Value
14–16	8.16 ± 6.24	0.081	7.66 ± 5.78	0.570 *	10.01 ± 5.72	0.015 *
17–22	8.55 ± 6.42		7.85 ± 6.01		10.53 ± 5.97	
Gender						
Male	6.85 ± 5.94	0.001 *	5.91 ± 5.25	* 0.001	8.14 ± 5.56	0.001 *
Female	9.06 ± 5.98		8.64 ± 6.31		11.28 ± 5.7	
School Grade						
1–3 semester group	8.28 ± 6.23	0.031	7.83 ± 5.89	0.669	10.05 ± 5.75	0.123
4–6 semester group	8.41 ± 6.44		7.66 ± 5.89		10.49 ± 5.95	
COVID-19 Infection						
Yes	10.06 ± 6.62	0.001 *	10.05 ± 6.07	* 0.001	12.25 ± 5.85	0.001 *
No	8.19 ± 6.28		7.54 ± 5.83		10.08 ± 5.81	
Family Members infected						
Yes	8.20 ± 5.88	0.002	8.6 ± 6.28	0.001	10.68 ± 5.74	0.001
No	7.15 ± 5.85		8.01 ± 6.379		9.70 ± 5.937	
Family COVID-19 survivor						
Yes	8.47 ± 6.25	0.006 *	8.09 ± 5.92	0.020 *	10.6 ± 5.77	0.103 *
No	9.79 ± 6.51		8.97 ± 5.67		11.30 ± 5.55	
Type of family						
Nuclear Family	7.75 ± 6.216		7.21 ± 5.76		9.73 ± 5.820	
Extended Family	8.93 ± 6.35	0.001 †	8.57 ± 6.08	0.001 †	10.95 ± 5.77	0.001 †
Single Parent Family	9.14 ± 6.45		8.23 ± 5.91		10.8 ± 5.83	
Others	10.27 ± 6.32		9.21 ± 5.97		11.86 ± 5.84	
Working mother						
Yes	8.60 ± 6.42	0.004	8.02 ± 5.97	0.001	10.47 ± 5.86	0.006
No	7.87 ± 6.13		7.25 ± 5.71		9.87 ± 5.80	
Working father						
Yes	8.26 ± 6.31	0.036	7.68 ± 5.88	0.051	10.22 ± 5.82	0.24
No	9.04 ± 6.46		8.36 ± 5.98		10.61 ± 6.04	

Notes: *: p-values were obtained by Student’s *t*-Test, †: *p-*values were obtained with Kruskal Wallis test.

**Table 4:** DASS-21 severity distribution between the overall sample, age groups, gender, school grade, family members with COVID-19, COVID-19 survivors, working mothers, working fathers, and type of families.

		Normal	Mild	Moderate	Severe	Extremely Severe
Total sample	D	1902 (61.1%)	431 (13.8%)	673 (21.6%)	106 (3.4%)	---
	A	1655 (53.2%)	321 (10.3%)	639 (20.5%)	390 (12.5%)	107 (3.4%)
	S	2262 (72.7%)	549 (17.6%)	301 (9.7%)	---	---
Age groups						
14–16	D	1019 (62.3%)	1019 (62.3%)	1019 (62.3%)	1019 (62.3%)	1019 (62.3%)
	A	229 (14%)	229 (14%)	229 (14%)	229 (14%)	229 (14%)
	S	335 (20.5%)	335 (20.5%)	335 (20.5%)	335 (20.5%)	335 (20.5%)
17–22	D	883 (59.8%)	229 (14%)	335 (20.5%)	52 (3.2%)	---
	A	775 (52.5%)	145 (9.8%)	311 (21.1%)	190 (12.2%)	56 (3.8%)
	S	1040 (70.4%)	271 (18.3%)	166 (11.2%)	---	---
Gender						
Male	D	713 (70.5%)	123 (12.2%)	150 (14.8%)	25 (2.5%)	---
	A	671 (66.4%)	86 (8.5%)	174 (17.2%)	61 (6%)	19 (1.9%)
	S	860 (85.1%)	97 (9.6%)	54 (5.3%)	---	---
Female	D	1189 (56.6%)	308 (14.7%)	523 (24.9%)	81 (3.9%)	---
	A	984 (46.8%)	235 (11.2%)	465 (22.1%)	329 (15.7%)	88 (4.2%)
	S	1402 (66.7%)	452 (21.5%)	247 (11.8%)	---	---
School Grade						
1st–3rd semester	D	1024 (61.8%)	234 (14.1%)	338 (20.4%)	62 (3.7%)	---
	A	897 (53.5%)	174 (10.4%)	334 (19.9%)	207 (12.4%)	64 3.8%)
	S	1228 (74.1%)	287 (17.3%)	143 (8.6%)	---	---
4th–6th semester	D	878 (60.4%)	197 (13.5%)	335 (23%)	44 (3%)	---
	A	776 (60.4%)	147 (11.4%)	305 (23.8%)	183 (14.3%)	43 (3%)
	S	1034 (71.11%)	262 (18%)	158 (10.9%)	---	---
COVID-19 Infection						
Yes	D	132 (52.4%)	31 (12.3%)	74 (29.4%)	15 (6%)	---
	A	94 (37.3%)	29 (11.5%)	61 (24.2%)	51 (20.2%)	17 (6.7%)
	S	152 (60.3%)	57 (22.6%)	43 (17.1%)	---	---
No	D	1170 (61.9%)	400 (14%)	599 (20.9%)	91 (3.2%)	---
	A	1561 (54.6%)	292 (10.2%)	578 (20.2%)	339 (11.9%)	90 (3.1%)
	S	2262 (72.7%)	549 (17.6%)	301 (9.7%)	---	---
Family Members with COVID-19						
Yes	D	1063 (60.1%)	253 (14.3%)	385 (21.8%)	68 (3.8%)	---
	A	889 (50.3%)	190 (10.7%)	376 (21.3%)	242 (13.7%)	72 (4.1%)
	S	1249 (70.6%)	340 (19.2%)	180 (10.2%)	---	---
No	D	839 (61.1%)	178 (13.3%)	288 (21.4%)	38 (2.8%)	---
	A	766 (57%)	131 (9.8%)	263 (19.6%)	148 (11%)	35 (2.6%)
	S	1013 (75.4%)	209 (15.6%)	121 (9%)	---	---
COVID-19 survivors						
Yes	D	930 (61.2%)	216 (14.2%)	320 (21.2%)	54 (3.6%)	---
	A	784 (51.6%)	154 (10.1%)	315 (20.7%)	207 (13.6%)	60 (3.9%)
	S	1075 (70.7%)	294 (19.3%)	151 (9.9%)	---	---
No	D	108 (49.3%)	36 (16.4%)	62 (28.3%)	13 (5.9%)	---
	A	93 (42.5%)	31 (14.2%)	52 (23.7%)	34 (15.5%)	9: (4.1%)
	S	151 (68.9%)	40 (18.3%)	28 (12.8%)	---	---
Working Mother						
Yes	D	1197 (59.3%)	284 (14.1%)	457 (22.6%)	81 (4%)	---
	A	1029 (51%)	208 (10.3%)	433 (21.4%)	273 (13.5%)	76 (7.1%)
	S	1433 (71%)	381 (18.9%)	205 (10.2%)	---	---
No	D	705 (64.5%)	147 (13.4%)	216 (19.8%)	25 (2.3%)	---
	A	626 (57.3%)	113 (10.3%)	206 (18.8%)	117 (10.7%)	31 (2.8%)
	S	829 (75.8%)	168 (15.4%)	96 (8.8%)	---	---
Working Father						
Yes	D	1711 (61.6%)	386 (13.9%)	591 (21.3%)	89 (3.2%)	---
	A	1501 (54.1%)	283 (10.2%)	591 (19.8%)	89 (12.6%)	95 (3.4%)
	S	2027 (70.1%)	484 (19.4%)	266 (9.6%)	---	---
No	D	191 (57%)	45 (13.4%)	82 (24.5%)	17 (5.1%)	---
	A	154 (46%)	38 (11.3%)	82 (26.9%)	17 (12.2%)	12 (3.6%)
	S	235 (70.1%)	65 (19.4%)	35 (10.5%)	---	---
Type of family						
Nuclear Family	D	1166 (65%)	228 (12.7%)	358 (19.9%)	43 (2.4%)	---
	A	1038 (57.8%)	175 (9.7%)	339 (18.9%)	191 (10.6%)	52 (2.9%)
	S	1365 (76%)	269 (15%)	161 (9%)	---	---
Extended Family	D	345 (56%)	105 (17%)	139 (22.6%)	27 (4.4%)	---
	A	281 (45.6%)	74 (12%)	139 (22.6%)	89 (14.4%)	33 (5.4%)
	S	428 (69.5%)	118 (19.2%)	70 (14.4%)	---	---
Single Parent Family	D	324 (56.8%)	81 (14.2%)	134 (23.5%)	31 (5.4%)	---
	A	282 (49.5%)	56 (9.8%)	130 (22.8%)	85 (14.9%)	17 (3%)
	S	389 (68.2%)	129 (22.6%)	52 (9.1%)	---	---
Others	D	67 (51.1%)	17 (13%)	42 (32.1%)	5 (3.8%)	---
	A	54 (41.2%)	16 (12.2%)	31 (23.7%)	25 (19.1%)	5 (3.8%)
	S	80 (61.1%)	33 (25.2%)	18 (13.7%)	---	---

Notes: D: depression, A: anxiety, S: stress. --- no participants.

## Data Availability

The raw data supporting the conclusions of this article will be made available by the authors, without undue reservation.
